# Case Report: Homozygous *DNAJC3* Mutation Causes Monogenic Diabetes Mellitus Associated With Pancreatic Atrophy

**DOI:** 10.3389/fendo.2021.742278

**Published:** 2021-09-24

**Authors:** Saud Alwatban, Haifa Alfaraidi, Abdulaziz Alosaimi, Iram Alluhaydan, Majid Alfadhel, Michel Polak, Angham Almutair

**Affiliations:** ^1^ College of Medicine, King Saud bin Abdulaziz University for Health Sciences, King Abdulaziz Medical City, Ministry of National Guard Health Affairs (MNG-HA), Riyadh, Saudi Arabia; ^2^ King Abdullah International Medical Research Centre (KAIMRC), Riyadh, Saudi Arabia; ^3^ Department of Pediatrics, King Abdullah Specialized Children’s Hospital, King Abdulaziz Medical City, Ministry of National Guard Health Affairs (MNG-HA), Riyadh, Saudi Arabia; ^4^ Medical Imaging Department, King Abdulaziz Medical City, Ministry of National Guard Health Affairs (MNG-HA), Riyadh, Saudi Arabia; ^5^ Genetics and Precision Medicine department, King Abdulaziz Medical City, Ministry of National Guard Health Affairs (MNG-HA), Riyadh, Saudi Arabia; ^6^ Medical Genomics Research Department, King Abdullah International Medical Research Centre (KAIMRC), King Abdulaziz Medical City, Ministry of National Guard Health Affairs (MNG-HA), Riyadh, Saudi Arabia; ^7^ Pediatric Endocrinology, Gynecology, and Diabetology Department, Necker University Children’s Hospital, Assistance Publique-Hôpitaux de Paris, IMAGINE Institute affiliate, INSERM U1163; INSERM U1016, Université de Paris, Paris, France

**Keywords:** *DNAJC3*, diabetes, monogenic diabetes, case report, pancreatic atrophy, neurodegeneration, short stature, hypothyroidism

## Abstract

**Introduction:**

DNAJC3, abundant in the pancreatic cells, attenuates endoplasmic reticulum stress. Homozygous *DNAJC3* mutations have been reported to cause non-immune juvenile-onset diabetes, neurodegeneration, hearing loss, short stature, and hypothyroidism.

**Case Description:**

We report a case of homozygous *DNAJC3* mutation in two siblings of a consanguineous family. A 3-year-old boy presented with short stature and a thyroid nodule. Laboratory findings confirmed hypothyroidism. Subsequently, levothyroxine was administered. Growth hormone (GH) stimulation test results were within the normal limits. His stature was exceedingly short (80.5 cm) (−3.79 SDS). The patient developed sensorineural hearing loss at age 6 years; his intellectual functioning was impaired. Recombinant Human Growth Hormine (rhGH) treatment was postponed until the age of 6.9 years due to a strong family history of diabetes. At age 9 years, he developed an ataxic gait. Brain magnetic resonance imaging (MRI) revealed neurodegeneration. The patient developed diabetes at the age of 11 years—5 years after the initiation of rhGH treatment. Tests for markers of autoimmune diabetes were negative. Lifestyle modification was introduced, but insulin therapy was eventually required. Whole-exome-sequencing (WES) revealed a homozygous *DNAJC3* mutation, which explained his clinical presentation. MRI revealed a small, atrophic pancreas. At the age of 17, his final adult height was 143 cm (−4.7 SDS). His elder brother, who had the same mutation, had a similar history, except that he had milder ataxia and normal brain MRI finding at the age of 28 years.

**Conclusion:**

We propose that *DNAJC3* mutation can be considered as a cause of maturity onset diabetes of the young. Patients with *DNAJC3* mutations may possess a small atrophic pancreas.

## Introduction

Diabetes mellitus (DM), one of the major public health challenges (1), is classified into type 1 diabetes (T1D), type 2 diabetes (T2D), gestational diabetes, and diabetes due to other causes, such as monogenic diabetes, exocrine pancreatic insufficiency, and diabetes secondary to mitochondrial disease (2). Typically, T1D occurs due to an autoimmune process that destroys the pancreatic β-cells, resulting in absolute insulin deficiency (3); T1D accounts for 75%, while T2D accounts for 15% of diabetes cases in pediatrics (4). Its incidence has increased in recent years due to the obesity epidemic (5–7). T2D is mainly caused by insulin resistance, resulting from poor insulin secretion leading to relative insulin deficiency (8).

In individuals with diabetes whose clinical manifestations cannot be categorized as either T1D or T2D, other causes of diabetes should be considered. For example, mitochondrial disease is considered in patients with DM accompanied by deafness, neurodegeneration, or optic nerve atrophy (9). Mitochondrial function is essential for normal insulin secretion and pancreatic response (10), with mitochondrial dysfunction leading to insulin resistance and pancreatic β-cell dysfunction (11). Additionally, mitochondria play a central role in aging-related neurodegenerative disorders as they are essential regulators of cell death—a key feature of neurodegeneration (12, 13). Monogenic diabetes is another important cause of diabetes in youth, accounting for 2–6% of all cases (14, 15). Maturity-onset diabetes of the young (MODY), one of the common causes of monogenic diabetes, is suspected when there is a family history of diabetes with an autosomal dominant pattern of inheritance and onset before 25 years old (16). Mutations in genes that regulate β-cell function have been identified as the cause of monogenic diabetes (17–21). Rare mutations, such as the *DNAJC3* mutation, which is associated with DM and multisystemic neurodegeneration, have been described recently (22–25). Herein, we report a familial case of *DNAJC3* mutation manifesting as juvenile-onset DM, hypothyroidism, multisystemic neurodegeneration, short-stature, and sensorineural hearing loss (SNHL). This case associates the previously described phenotype of *DNAJC3* mutation with the new finding of pancreatic fibrosis and atrophy. To the best of our knowledge, this is the first case of a *DNAJC3* mutation reported in the Arab region.

This study was approved by the IRB of KAIMRC. Written informed consent was obtained from the patients and their guardians for publication.

## Case Description

We report a 2-year-10-month-old boy (patient A) who was referred to the endocrinology clinic due to short-stature, with no symptoms suggestive of chronic illness. He had a small body, triangular face, and deep-set eyes. His growth parameters were as follows: height, 80.5 cm (−3.7 SDS); weight, 10.3 kg (−2.79 SDS); and head circumference, 45.5 cm (-2.45 SDS). Patient A was born prematurely at 32 weeks’ gestation *via* normal vaginal delivery (birth weight, 2 kg, +0.59 SDS and birth length 42 cm, +0.8 SDS) (26). The patient was appropriate for gestational age.

**Figure 1:**
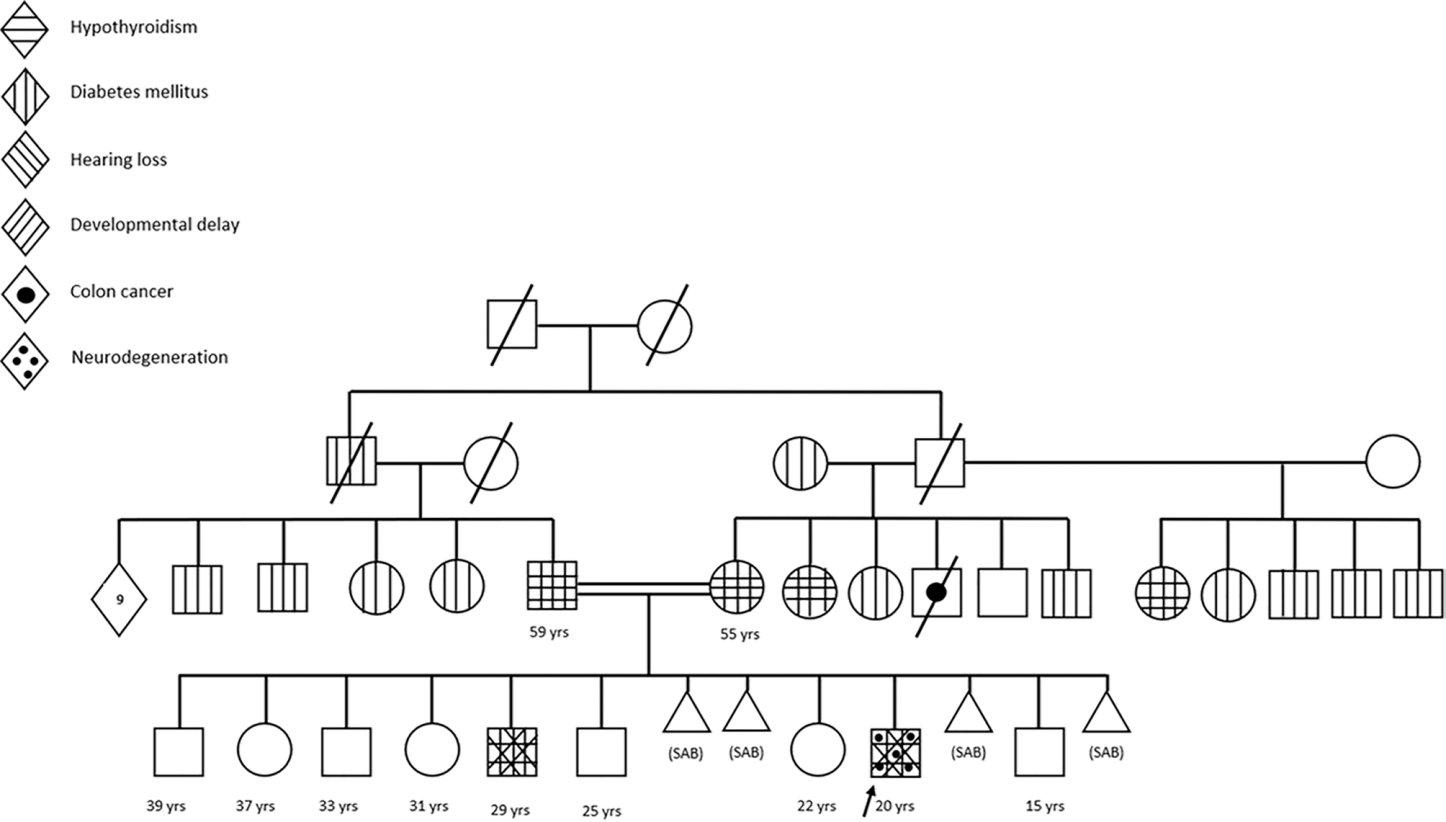
Family pedigree of patients A and B.

Regarding family history, the mother’s height was 151 cm, and the father’s height was 165 cm, with a mid-parental height (MPH) of 164.5 cm (−1.62 SDS). The parents were first-degree cousins. Multiple members of the maternal family had DM (**Figure 1**). Of note, the patient’s elder brother (patient B) was also followed up for short-stature and was known to have DM and hypothyroidism (described further below).

Initial investigation of patient A revealed a thyroid-stimulating hormone (TSH) level of 27.8 mIU/L and free thyroxine (FT4) level of 6.7 pmol/L (**Table 1**). The thyroid uptake was normal. Thyroid antibody tests were negative, and neonatal cord blood TSH screening result was normal. Based on the initial results, patient A was administered 25 µg of levothyroxine daily (2.5 mcg/kg/day). Other laboratory investigations, including liver function tests, complete blood count, erythrocyte sedimentation rate, celiac screening, bone profile, and parathyroid hormone levels, were within the normal limits. Additionally, the skeletal survey was normal, and chromosomal analysis showed a 46, XY karyotype with no structural abnormalities.

**Table 1:** Thyroid function tests in individuals with homozygous *DNAJC3* mutation.

Test	Present Study		Bublitz et al. (25)	Synofzik et al. (22)				Ozon et al. (23)		Lytrivi et al. (24)	
	Patient a	Patient b	Patient a	Patient a	Patient b	Patient c	Patient d	Patient a	Patient b	Patient a	Patient b
**TSH**	27.8 (0.5–5.0)	28.2 (0.5–5.0)	4.9 (0.5–5.0)	2.93 (0.5–5.0)	20.9 (0.5–5.0)	Transient elevation	Transient elevation	22.0 (0.45–4.20)	11.2 (0.45–4.20)	Elevated	High
**FT4**	6.7 (9.0–19.0)	12.0 (9.0–19.0)	14.2 (11.6–20.6)	10.95 (11.6–20.6)	9.65 (11.6–20.6)	–	–	10.5 (12.0–22.0)	11.1 (12.0–22.0)	Normal	Low
**Thyroid function tests after treatment initiation**											
**TSH**	90.7 (0.5–5.0)	17.1 (0.5–5.0)	–	–	–	–	–	0.012 (0.45–4.20)	3.9 (0.45–4.20)	–	–
**FT4**	6.4 (9.0–19.0)	10.8 (9.0–19.0)	–	–	–	–	–	15.8 (12.0–22.0)	11.2 (12.0–22.0)	–	–
**TSH**	1.07 (0.5–5.0)	5.7 (0.5–5.0)	–	–	–	–	–	–	–	–	–
**FT4**	12.1 (9.0–19.0)	10.3 (9.0–19.0)	–	–	–	–	–	–	–	–	–

TSH, thyroid stimulating hormone, values reported in mIU/L (normal range: 0.35–4.94); FT4, free thyroxine, values reported in pmol/L (normal range: 9.00–19.00); -, not available.

The growth hormone (GH) stimulation test (Clonidine), conducted after thyroid function normalization, showed a normal peak at 15.3 ng/mL (normal >10 ng/ml). Serum IGF-1 was 132 ng/ml (normal 52–297 ng/ml). On subsequent follow-up at the age of 4.5 years, the patient’s height was 85 cm (−4.5 SDS), and growth velocity (GV) was suboptimal (2.5 cm/year, −3.1 SDS). rhGH therapy was deferred to a later age due to the risk of insulin resistance and the significant family history of DM (**Figure 1**). Of note, the patient’s elder brother (patient B) had been treated with rhGH therapy and subsequently developed diabetes. However, due to persistently low GV and short stature, rhGH therapy was initiated at a height 96.5 cm (−4.4 SDS) with a small dose (0.015 mg/kg/day) for patient A at the age of 6.9 years. Bone age was normal for chronological age, and hemoglobin A1c (HbA1c) level was 4.6% (<5.7) prior to rhGH therapy. GV during 1^st^ year of treatment was 3.9 cm (−2.1 SDS); therefore the rhGh dose was increased to 0.025 mg/kg/day. HbA1c levels were frequently assessed during rhGH therapy due to the strong family history of DM. Serum IGF-1 was 154 ng/ml (57–316) on rhGH therapy. Consequently, rhGH was discontinued at 10.10 years due to poor response (height 111 cm, −4.6 SDS), and elevated HbA1c (7.6%). Physical examination revealed normal body mass index (BMI), and absence of acanthosis nigricans. He was prepubertal at that time. Insulin and C-peptide levels were detectable. Autoimmune diabetes markers (glutamic-acid decarboxylase, islet-cell antibodies, and insulin antibodies) were absent; the tests were repeated multiple times and remained negative. He was managed with lifestyle modifications, eventually requiring insulin therapy 3 years later.

**Figure 2:**
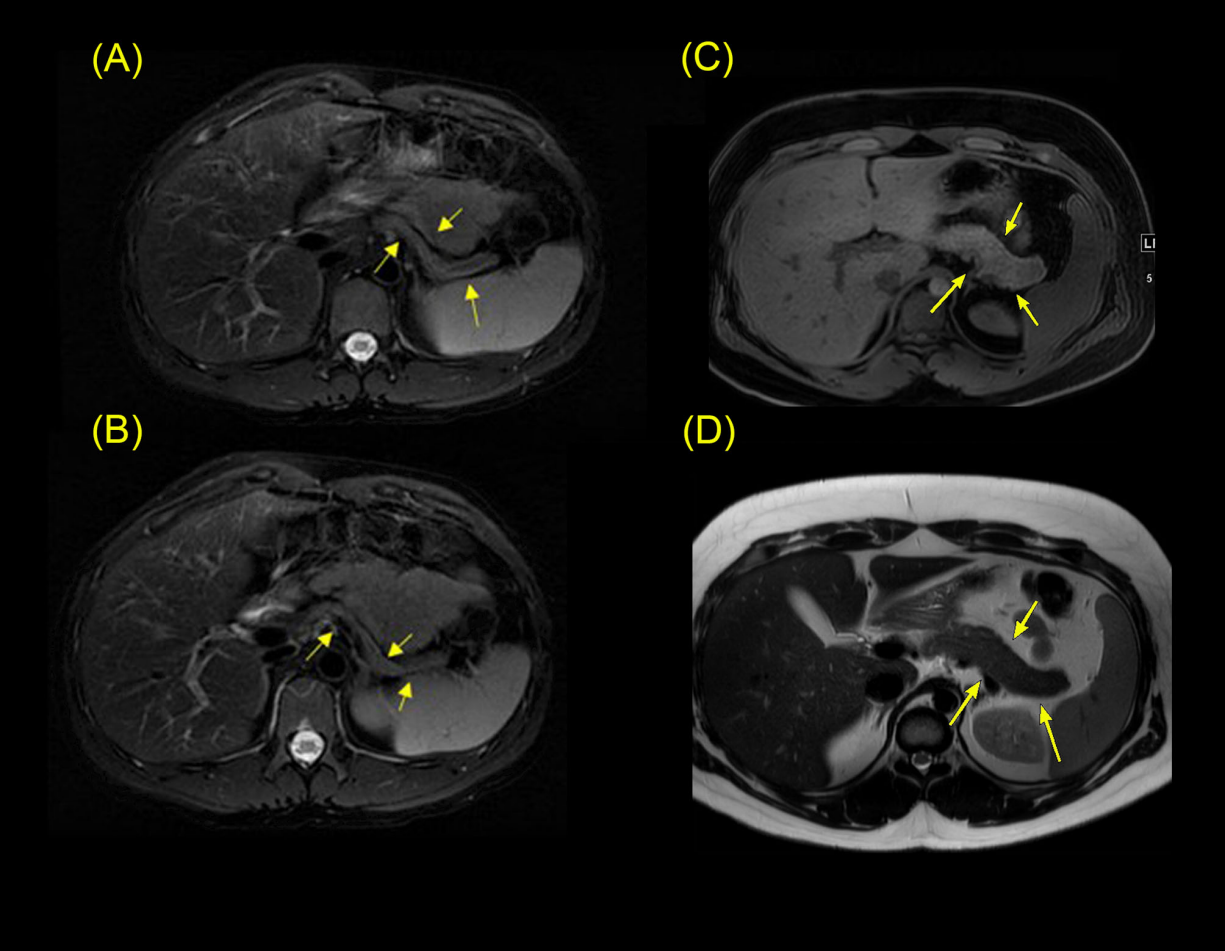
Pancreatic MRI scan of the index patient (patient A) and control patient. The MRI scan in **(A, B)** showing a small atrophic pancreas of the index patient (patient A). Images clearly show global parenchymal atrophy of the pancreas. The pancreatic parenchyma appears thin with heterogeneous low T1 signals, indicating fibrosis. The MRI scan in **(C, D)** are axial T2 weighted MRI images of a control case, a 26-year-old male with type 1 DM. The images **(C, D)** show a normal volume of the pancreas.

At 13 years old, the patient’s glycemic control deteriorated, HbA1c level rising to 9.8% with DM symptoms. This coincided with the progression of puberty. At that time, he was at Tanner stage III for pubic hair and testicle volume. Basal-bolus insulin therapy was initiated (0.5 unit/kg/day). His HbA1c dropped to 6.9% and 7.1% after 3 and 12 months, respectively. His final height was 143 cm (-4.7 SDS, corrected for MPH –3.9). Finally, pancreatic exocrine function, assessed by levels of lipase and vitamins A, D, E, and K, was normal. MRI of the pancreas revealed a small, shrunken, and atrophic pancreas, with signs of fibrosis at the age of 18 years (**Figure 2**). Of note, there was no history of neonatal hyperinsulinemic hypoglycemia.

From a neurodevelopmental point of view, patient A showed a delay in the attainment of language, with only two-word sentences at 5 years and delay of other milestones. Audiometry confirmed bilateral SNHL. Speech evaluation revealed good perception but poor expressive language, with severe impairment in intelligible speech production. Although he was 6 years old, he could not attend school. His intellectual function was reported as mild-to-moderate intellectual disability, with significant academic delay and an inability to proceed to intermediate school.

At 9 years of age, gait abnormalities started to develop, requiring multiple visits to the emergency department due to frequent falls. His gait was unsteady, with high heel steps and progressive deterioration. Rheumatological causes were excluded based on clinical evaluations and laboratory tests. Neurological examination showed increased deep tendon reflexes, mild bilateral nystagmus, and atrophied lower limb muscles. Cerebellar examination revealed bilateral dysmetria in the upper extremities. Brain and spine MRI demonstrated bilateral subcortical white matter intensity with bilateral middle cerebellar peduncle involvement. Electromyography revealed prolongation over the tibial and ulnar nerves. His visual acuity was normal. Both atypical multiple sclerosis and mitochondrial disease were considered because of the patient’s progressive history. The former was excluded based on normal visual evoked potentials and negative antinuclear antibodies, while the latter was excluded due to normal results of muscle and skin biopsy, whole mitochondrial genome sequencing, respiratory chain enzymology, magnetic resonance spectroscopy, and urine amino acid levels.

Patient B, the elder brother of patient A, was initially evaluated at 5 years of age for short-stature and found to have subclinical hypothyroidism, requiring levothyroxine (2 mcg/kg/day). Serum TSH level was 28.5 mIU/L, while serum FT4 level was 12 pmol/L (**Table 1**), with normal thyroid uptake scan and negative thyroid antibodies. rhGH therapy was initiated at 8 years of age at a dose of 0.35 mg/kg/day; his initial HbA1c level was 5.9%.

Regarding development, patient B was hyperactive; a learning or intellectual disability and cognitive impairment were suspected as he had academic difficulties, requiring repetition of three school years. Audiometry confirmed bilateral SNHL at the age of 11 years; the karyotype was 46XY, and fragile X test was negative. Finally, pancreatic MRI showed a small-atrophic pancreas similar of his brother; pancreatic exocrine function was normal, with no symptoms suggestive of malabsorption.

rhGH therapy was intermittently continued for 6 years with poor response. At 14 years of age, patient B developed DM with a normal BMI. He was at Tanner stage III for pubic hair and testicle volume. rhGH treatment was discontinued, and metformin was initiated in addition to lifestyle modifications. One year later, the patient required insulin due to deterioration of his glycemic control (HbA1c, 13%). Similar to the index patient, he developed a mild form of ataxia. Neurological examination revealed abnormal cerebellar signs in the form of bilateral dysmetria in the upper extremities, dysdiadochokinesia, and intention tremor. Audiometry confirmed bilateral SNHL. However, a brain MRI performed for patient B at 28 years of age showed no signs of neurodegeneration. His final height was 147 cm (–4.01 SDS, corrected for MPH –3.3 SDS).

Owing to the progressive disease nature, and the concerning brain MRI changes in patient A, WES was performed. The result showed a previously reported homozygous pathogenic variant in *DNAJC3* [NM_006260.5:c.1177C>T;p.(Arg393Ter)], by Lytrivi et al. (24), with an autosomal recessive mode of inheritance. Both parents are carriers, which explained the patient’s clinical presentation. Other family members were analyzed for segregation, and it was shown to be well segregated with the phenotype. The variant was identified as pathogenic/likely pathogenic in several international databases like ClinVar, Varsome and gnomAD. Furthermore, the mutation is extremely rare in various populations, with a mean allele frequency (MAF) of less than 1%. In silico parameters like BayesDel addAF, DANN, EIGEN, FATHMM-MKL, and MutationTaster predict this variant as disease causing/damaging. Subsequently, WES was performed for patient B, and the presence of the same mutation was confirmed.

## Discussion

To the best of our knowledge, this is the first reported case of *DNAJC3* mutation reported in the Arab region. *DNAJC3* functions as a co-chaperone of BiP, which promotes the proper folding of proteins in the endoplasmic reticulum (ER) by binding to amino acid segments of unfolded proteins (27). ER stress induces *DNAJC3* expression, which then inhibits eukaryotic initiation factor−2 signaling and reduces the unfolded protein response, thereby decreasing apoptosis (28). While *DNAJC3* is present in all tissues, it is predominantly found in the pancreatic cells, including β-cells, and hepatocytes (29). Since *DNAJC3* plays a vital role in attenuating the ER stress, defective *DNAJC3* leads to the activation of cellular apoptosis, with loss of pancreatic β-cells and decreased insulin secretion (30). This is evidenced by the hyperglycemia observed in *DNAJC3*-null mice (31). Interestingly, *DNAJC3*-null mice also showed reduced body weight (31). Defects in other genes that mediate ER stress response have also been associated with widespread neurodegeneration (32–35), potentially explaining the neurodegenerative symptoms observed in individuals with *DNAJC3* mutations. For example, in Wolfram syndrome, which consists of optic nerve atrophy, SNL and insulin-dependent DM, abnormal ER stress regulation and signaling occurs with resulting apoptosis (36, 37).

Synofzik et al. first described two families with *DNAJC3* mutations in 2014 (22). Five individuals were described, all of whom had juvenile-onset diabetes and a combination of various neurodegenerative symptoms, including SNHL, ataxia, abnormal nerve conduction, and cognitive deficits (**Table 2**). Short-stature and low weight were also observed. The phenotype of *DNAJC3* mutations was later expanded to include hypothyroidism based on a case reported by Bublitz et al. (25). The authors described a 20-year-old female with juvenile-onset diabetes, SNHL, cognitive deficits, ataxia, and sensorimotor neuropathy, with hypothyroidism and slightly elevated TSH levels that improved with thyroid hormone replacement.

**Table 2:** Characteristics of individuals with homozygous *DNAJC3* mutations.

Patient	Present Study		Synofzik et al. (22)		Bublitz et al. (24)	Ozon et al. (23)		Lytrivi et al. (24)	
	a	b							
***DNAJC3* variant**	c.1177C>T (p.R393X)		c.580C>T (p.Arg194*)	Deletion of exons 6–12(p.)?	c.580C>T, (p.Arg194)	(c.393+2T>G,NM_006260.4)	(c.393+2T,C, NM_006260.4)	(c.1036C>T, p.R346*) Compound Het	(c.1177C>T, p.R393*)
**Consanguinity**	+		–	+	–	+	+	–	+
**Ethnicity**	Middle Eastern		Turkish	Turkish	Turkish	Presumed Turkish	Presumed Turkish	Armenian	Algerian
**Number of members affected**	2		3	2	1	1	1	1	1
**Sex**	Male	Male	Male (a, b): 2	Female (a, b)	Female	Male	Female	Female	Male
			Female (c): 1						
**Final adult height, cm (Z-score)**	143 (−4.7)	147 (−4.1)	a: 152 (−3.44)	a: 136 (−4.18)	149 (−2.20)	NA*	NA*	143 (−3)	150.5 (−4)
			b: 156 (−2.89)	b: 143 (−3.12)					
			c: 145 (−2.81)						
**Short stature**	+	+	+ in 2/3	+	+	+	+	+	+
**Adult weight, kg (Z-score)**	31.4 (7.44)	51 (−2.35)	a: 45 (−3.45)	a: 39 (−3.31)	42 (−2.53)	NA*	NA*	43	36
			b: 49 (−2.69)	b: 39 (−3.31)					
			c: 38 (−3.60)						
**BMI in kg/m^2^ (Z-score)**	17.49 (−2.65)	23.60 (0.18)	a: 19.50 (−1.44)	a: 21.10 (−0.19)	18.90 (−1.07)	17.10 (1.00)	17.40 (0.48)	21.0 (−3.6)	15.9 (−5.6)
			b: 20.10 (−1.15)	b: 19.10 (−0.98)					
			c: 18.10 (−1.49)						
**Hypothyroidism**	+	+	+ in 2/3	NA	+	+	+	+	+
**Hypoacusis**	+	+	+	+ in 1/2	+	+	+	+	+
**Age during onset of hypoacusis, years**	6	8.5	a: 6	a: 2	7	6.5	5	6	NA
			b: 14	b: NA					
			c: 27						
**Cognitive deficits**	+	+	+	NA	+	+	+	–	NA
**Ataxia**	+	+	+	+	+	+	+	+	NA
**Age during onset of ataxia, years**	9	NA	a: 6	a: 2	15	15.5	3	Neonate	NA
			b: 19	b: 11					
			c: 34						
**Sensorimotor neuropathy**	+	–	+	+	+	+	+	+	NA
**DM**	+	+	+	+	+	+	+	+	+
**Age during onset of DM, years**	11	14	a: 18	a: 14	19	15.5	13.8	12	16
			b: 15	b: 11					
			c: 18						
**Abnormal brain and spine MRI findings**	+	–	+	+	+	+	+	+	NA
**Abnormal pancreas MRI findings**	+	+	NA	NA	NA	NA	NA	NA	NA

*Final adult height and weight was not achieved at date of publication.

BMI, body mass index; DM, diabetes mellitus; NA, not available

Z-scores and SDS are based on Centers for Disease Control and Prevention growth charts 2000 (38).

Of the five individuals reported by Synofzik et al. (22), two required thyroid replacement, while the other two had transient TSH elevations. Two unrelated children with *DNAJC3* mutations have also been recently described by Ozon et al. as having diabetes, neurodegenerative symptoms, and hypothyroidism requiring thyroid replacement (23). Furthermore, two unrelated patients were recently reported to have the same symptoms, including hypothyroidism (24). The presence of hypothyroidism in the siblings we reported herein, supports hypothyroidism should be considered a feature of *DNAJC3* mutations. However, the etiopathogenesis of hypothyroidism associated with *DNAJC3* mutations remains unclear. We observed various hormonal patterns in thyroid function tests performed in individuals with homozygous *DNAJC3* mutations (**Table 1**). An elevated TSH level of 90.7 mIU/L in patient A (**Table 1**) suggests primary hypothyroidism as the most likely cause, which may reflect a vital role of the *DNAJC3* gene in thyroid hormone synthesis.

Juvenile-onset DM is a frequent manifestation of homozygous *DNAJC3* mutations, with the onset of hyperglycemia occurring between 11 and 19 years of age (22–25). In such cases, DM is characterized by a lack of pancreatic autoimmunity markers, an insidious onset, and normal-to-low BMI without signs of insulin resistance (22–25); these presentations limit the diagnosis as either T1D or T2D, pointing to another cause. Of note, anti-glutamic acid decarboxylase (anti-GAD) antibody tests were positive in one of the individuals described by Synofzik et al. (23); however, anti-islet tyrosine phosphatase 2 antibody tests were negative. Moreover, less than half of individuals with isolated positive GAD-antibodies develop diabetes over an 18-year period (34).

The two cases reported by Ozon et al. had a history of hyperinsulinemic hypoglycemia, and both required diazoxide (24). This reflects abnormal β-cell function and is in keeping with other causes of monogenic diabetes, such as *HNF1A* and *HNF4A* mutations, wherein hyperinsulinemic hypoglycemia occurs in early life with the development of diabetes at an older age (39–41). The detectable levels of endogenous insulin observed in patient A have also been described in insulin gene mutations that lead to β-cell ER stress and eventually apoptosis and resulting in higher insulin levels than in autoimmune DM (42, 43).

Structural anomalies of the pancreas are common in patients with monogenic diabetes (44). Pancreatic anomalies can present as atrophy, aplasia, agenesis, and lipomatosis, which are typical in certain types of MODY and neonatal diabetes (45, 46). *HNF1A*, *HNF1B*, and *CEL*-MODY gene mutations are known to cause pancreatic hypoplasia (47, 48). In our two cases, MRI showed hypoplasia and atrophy of the pancreas. These findings support the involvement of *DNAJC3* in pancreatic development. No previously described cases underwent pancreatic MRI and pancreatic anatomic features are not described in the literature for patients with *DNAJC3* mutation. This feature of the pancreas is to be added as a phenotype of the *DNAJC3* mutation. We suggest using imaging modalities to evaluate the structural changes of the pancreas in the early stages of suspected monogenic DM to characterize their progression.

The patients reported here, and the four described by Ozon et al. (23) and Lytrivi et al. (24) were administered rhGH therapy for 2-5 years. GH is a counterregulatory hormone, promoting insulin-antagonistic effects both in the liver and in peripheral tissues. While it has a mitogenic effect on β-cells; it causes hyperglycemia by stimulating glucose production through increased glycogenolysis and gluconeogenesis (49, 50). Additionally, GH decreases glucose uptake by adipose tissue (47). Therefore, the hyperglycemia developed was presumed to be related to rhGH therapy in the setting of limited insulin secretory capacity due to the β-cell’s genetic anomaly. However, hyperglycemia persisted or recurred in all six patients even after the discontinuation of rhGH therapy, indicating the hyperglycemia was, in fact, due to new-onset diabetes. We hypothesized rhGH accelerated hyperglycemia-induced ER stress (42), which facilitated β-cell loss in the setting of *DNAJC3* mutation and an inadequate response to ER stress. Frequent monitoring of HbA1c levels during rhGH therapy may have contributed to our earlier detection of hyperglycemia compared to the other reported cases of homozygous *DNAJC3* mutations.

It is unclear whether individuals with homozygous *DNAJC3* mutations have insufficient GH secretory capacity. The two patients we described herein showed a normal response on GH stimulation, as did the three patients reported by Bublitz et al. (25) and Lytrivi et al. (24). In the report by Ozon et al. (23), one patient had a sufficient GH response initially, with a subsequent suboptimal response, whereas the second patient had an initial suboptimal GH peak. However, rhGH therapy response was inadequate in all patients, which is unusual in true GH deficiency. Therefore, rhGH treatment should not be initiated in patients with *DNAJC3* mutations, given their increased risk of diabetes and inadequate response to rhGH therapy. The risk of hyperglycemia may outweigh the potential growth benefit.

Neurological manifestations are present in all patients, in varying degrees. The constellation of neurological symptoms includes SNHL, cognitive deficits, ataxia, and sensorimotor neuropathy (22–25). The loss of mitigation of the ER stress response is implicated in the pathogenesis of widespread neurodegeneration associated with the *DNAJC3* mutation. Other gene mutations leading to dysregulation of ER stress by affecting BiP co-chaperones or other transmembrane proteins have been associated with multisystemic neurodegenerative disorders, such as Marinesco-Sjögren syndrome and Wolfram syndrome (32–35). Notably, mitochondrial disease was considered a potential etiology in patient A and in previous reports of *DNAJC3* mutations due to overlapping clinical presentations of diabetes, hearing-impairment, and neurodegeneration. Therefore, we suggest *DNAJC3* mutations should be included in the differential diagnosis when investigating suspected mitochondrial disease.

Our knowledge of the homozygous *DNAJC3* mutation phenotypes is evolving. This condition should be suspected in youth presenting with juvenile-onset diabetes, ataxia, SNHL, progressive neurodegeneration, short stature, and hypothyroidism. Given early age of diabetes onset, absence of autoimmunity markers, lack of signs of insulin resistance, and imaging findings of pancreatic atrophy, we propose adding *DNAJC3* mutations to the list of mutations known to cause monogenic diabetes. rhGH therapy seems not effective in promoting growth acceleration, thus prescription should be discussed with the family considering available evidence.

## Patient Perspective

Patient A stated that he could not complete high school. He experiences difficulty in walking for long distances; this requires him to use a wheelchair. He does not experience mood swings. He requires his mother’s assistance in performing some of the outdoor daily activities.

Patient B reported he is a high school graduate coping with his condition with minimal effect on his quality of life. He has a simple job and is unmarried. He can drive a car, take care of himself as a mature adult, and has a good relationship with friends.

## Data Availability Statement

The raw data supporting the conclusions of this article will be made available by the authors, without undue reservation.

## Ethics Statement

The studies involving human participants were reviewed and approved by Institutional Review Board (IRB) at King Abdullah International Medical Research Center (KAIMRC), Riyadh, Saudi Arabia. Written informed consent to participate in this study was provided by the participants’ legal guardian/next of kin. Written informed consent was obtained from the individual(s), and minor(s)’ legal guardian/next of kin, for the publication of any potentially identifiable images or data included in this article.

## Author Contributions

SA collected the patient data. SA and HA wrote the manuscript. AbA contributed to the radiological parts. IA worked on the family pedigree. MA revised the genetic aspects of the study. MP revised the manuscript. AnA supervised the entire process, as well as revised and approved the manuscript. All authors contributed to the article and approved the submitted version.

## Funding

Open access publication fees will be funded by King Abdullah International Medical Research Center (KAIMRC), Riyadh, Saudi Arabia.

## Conflict of Interest

The authors declare that the research was conducted in the absence of any commercial or financial relationships that could be construed as a potential conflict of interest.

## Publisher’s Note

All claims expressed in this article are solely those of the authors and do not necessarily represent those of their affiliated organizations, or those of the publisher, the editors and the reviewers. Any product that may be evaluated in this article, or claim that may be made by its manufacturer, is not guaranteed or endorsed by the publisher.
