# Supplementary material for: Case Report: Homozygous DNAJC3 Mutation Causes Monogenic Diabetes Mellitus Associated With Pancreatic Atrophy
Source: Front Endocrinol (Lausanne). 2021 Sep 24;12:742278. doi: 10.3389/fendo.2021.742278 (PMC8497828; doi:10.3389/fendo.2021.742278)
Supplement: Supplementary file 1 [file DataSheet_1.pdf]

CARE case report guidelines

CARE Checklist of information to include when writing a case report

(cc) BY-NC-ND

Topic	Item	Checklist item description	Reported on Line
Title	1	The diagnosis or intervention of primary focus followed by the words "case report"	Title
Key Words	2	2 to 5 key words that identify diagnoses or interventions in this case report, including "case report"	23,24
Abstract (no references)	3a	Introduction: What is unique about this case and what does it add to the scientific literature?	27-29
	3b	Main symptoms and/or important clinical findings	30-44
	3c	The main diagnoses, therapeutic interventions, and outcomes	30-44
	3d	Conclusion—What is the main "take-away" lesson(s) from this case?	45,46
Introduction	4	One or two paragraphs summarizing why this case is unique (**may include references**)	49-80
Patient Information	5a	De-identified patient specific information.	82-88
	5b	Primary concerns and symptoms of the patient.	86-91,115,126-131
	5c	Medical, family, and psycho-social history including relevant genetic information	84-93,163-165
	5d	Relevant past interventions with outcomes	101,107,108,112
Clinical Findings	6	Describe significant physical examination (PE) and important clinical findings.	86-91,115,116,128-130,132-138
Timeline	7	Historical and current information from this episode of care organized as a timeline	85-173
Diagnostic Assessment	8a	Diagnostic testing (such as PE, laboratory testing, imaging, surveys).	97-105,115-119,122-125,12-129,135-147
	8b	Diagnostic challenges (such as access to testing, financial, or cultural)	108-110
	8c	Diagnosis (including other diagnoses considered)	86,101,115,128,130,132,138, 140,146
	8d	Prognosis (such as staging in oncology) where applicable	135-140,146
Therapeutic Intervention	9a	Types of therapeutic intervention (such as pharmacologic, surgical, preventive, self-care)	100,112,121
	9b	Administration of therapeutic intervention (such as dosage, strength, duration)	100,112,121
	9c	Changes in therapeutic intervention (with rationale)	112,121
Follow-up and Outcomes	10a	Clinician and patient-assessed outcomes (if available)	279-285
	10b	Important follow-up diagnostic and other test results	117,122,146,147,163,171
	10c	Intervention adherence and tolerability (How was this assessed?)	107,117,122,163
	10d	Adverse and unanticipated events	105-110,119,124,129
Discussion	11a	A scientific discussion of the strengths AND limitations associated with this case report	176-278
	11b	Discussion of the relevant medical literature **with references**.	176-278
	11c	The scientific rationale for any conclusions (including assessment of possible causes)	197-205
	11d	The primary "take-away" lessons of this case report (without references) in a one paragraph conclusion	266-273
Patient Perspective	12	The patient should share their perspective in one to two paragraphs on the treatment(s) they received	275-280
Informed Consent	13	Did the patient give informed consent? Please provide if requested	Yes ☑ No ☐
