# Supplementary material for: Case Report: Homozygous DNAJC3 Mutation Causes Monogenic Diabetes Mellitus Associated With Pancreatic Atrophy
Source: Front Endocrinol (Lausanne). 2021 Sep 24;12:742278. doi: 10.3389/fendo.2021.742278 (PMC8497828; doi:10.3389/fendo.2021.742278)
Supplement: Supplementary file 2 [file Image_1.pdf]

Figure 2: Brain MRI for Index patient (Patient A)

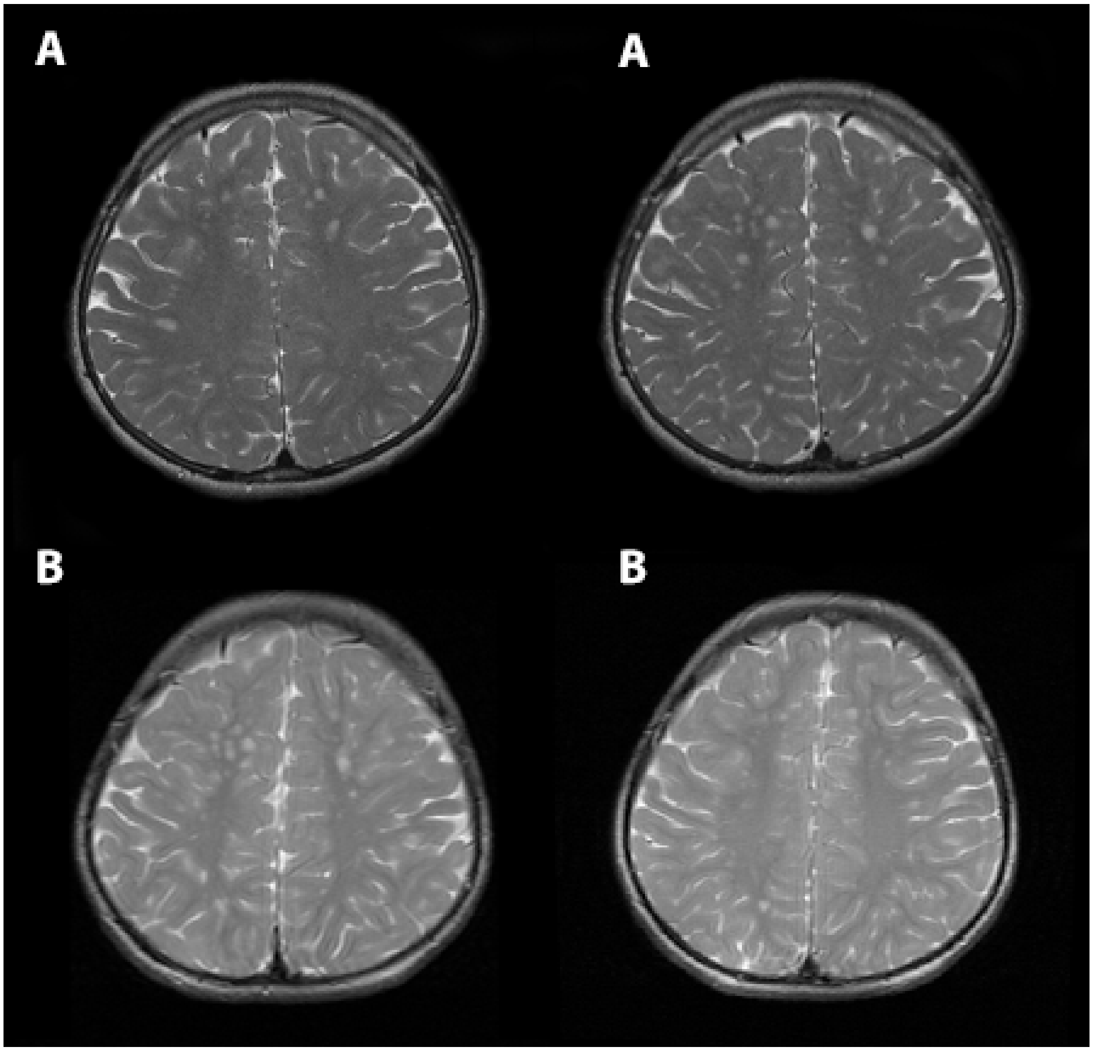

MRI A: at 13 years of age. MRI B: at 16 years of age. Images show multiple T2 subcortical white matter lesions within the cerebral hemispheres bilaterally, temporal lobes and brainstem. These foci demonstrated no enhancement post Gadolinium administration. There is subtle signal abnormality along bilateral middle cerebellar peduncles.
